# Child Life under Queen Victoria

**Published:** 1897-02-20

**Authors:** 


### Child Life under Queen Victoria.

### II.—TEXTILE FACTORIES: THE CHILDREN'S WRONGS.

The advent of machinery, of water-power, and afterwards of steam-power, changed the whole nature of the weaving trade. Formerly the weaver worked in his own home, assisted by his wife and children, and only a few had two or three looms and employed hired assistants. But with machine-power, which could work scores or hundreds of looms, concentration of labour in one spot became inevitable. The factory was built, often at first in some lonely dale, where a mountain stream gave the necessary power. Had steam been employed from the first, the factories would probably have been built in existing weavers' villages, which might to some extent have affected the development of the factory system, although no such difference in the power employed would have affected the case of the farmer-weaver, who tilled the land he had inherited from long generations of ancestors, and made his weaving only a by-work. But the weavers would not go to the lonely factory, and did not guess how independent of their skill the new monster had made the capitalist.

In the weaving of all ordinary fabrics there was now no operation left for human labour which, literally, a child could not do as well as a man. Better, indeed, for the first factories were small and overcrowded, and the small, lithe bodies of children and their nimble movements were really better adapted to the work and the conditions in which it was carried on than those of their seniors.

But where, if no families lived near the factories, did the children come from? To tell that is to recount one of Britain's blackest shames. The Poor Law Act of Elizabeth's reign, the foundation of all legislation on that subject, ordained that the guardians of the poor were to apprentice pauper children. It was probably intended that the children should be apprenticed within their own parish, that their subsequent labour might repay the cost of their previous maintenance. But when with the advent of the factory system came the unprecedented demand for children's labour, it was not hard to construe the Act differently. Masters and poor law officers communicated with each other on the subject, and when terms were fixed a batch of children would be sold into the slavery of the mills. Sometimes they were cajoled with promises of money and comfort, that they might be regarded as volunteers; but to such friendless creatures it was hardly necessary even to lie. They had no option but to accept their fate.

What that fate was, seems incredible in a country and a generation that with such good will abolished the slave trade. Well might Lord Shaftesbury plead, in the bitterness of his noble heart, that the nation which held forty-five hours of work a week to be enough for an adult negro should not condemn English children to labour for more than sixty-nine. Fourteen hours a day was the recognised average for work, but when trade was good often sixteen hours were wrought. The factories were small, and the machinery all unfenced, and children were allowed to clean the machines while they were in motion. Few completed their apprenticeship without scars; many were maimed for life. Lord Shaftesbury tells of a young girl who was injured by a machine, and whose employers stopped eighteenpence out of her scanty wages for the proportion of the week after the accident. True, she had not earned that eighteenpence, but it was an expensive piece of meanness for the employer. Lord Shaftesbury took up the case, brought it into a court of law, and the master had to pay £100 damages. But that was when things were beginning to mend, and public opinion was aroused. The air, moreover, was full of dust which the workers inhaled, laying the seeds of future disease, while what was called "wet spinning" caused chills that were equally favourable to the development of consumption. The children were lodged in dens where neither health nor decency were possible. Their food was scanty, and of the coarsest kind—porridge and black bread, with occasionally a little coarse bacon. It is a literal fact that these hapless children, like the prodigal of Scripture "were fain to fill their belly with the husks that the swine did eat." One of their number, Robert Blincoe, told in his autobiography how he and the other boys used to steal the pigs' meat till the animals grew shrewd enough to drag it through the dirt at once. Even their poor food they could not always get, for in their brief dinner hour—they had but forty minutes' rest in all their fourteen hours of work—the overseers would contrive to cheat them by putting the clock back while they were working, and setting it to the right time when they rested.

Besides this ordinary run of harsh treatment, the most brutal torments were inflicted by masters and overseers out of sheer malice, or as a punishment for trifling offences. Robert Blincoe's autobiography gives these in great detail; and, though his story might be regarded as prejudiced, it is confirmed by the testimony of others. He was beaten black and blue, but that was a trifle. He was suspended by the thumbs over the machinery, which, as he had to huddle his legs under him to prevent their being injured by the wheels, proved an ingenious and complex torture. His head was cut with rollers that were thrown at him; he was compelled to work with vices affixed to his nose or ears. He suffered more cruelties than we have space to detail, or than there is any need at this period to narrate; yet he declares that others were worse treated than himself. An essential member of the factory staff was the smith, who rivetted fetters on apprentices—slaves!—who were suspected of any intention of running away. One girl tried to escape from thraldom by throwing herself into the mill-dam. She was rescued, and, fortunate creature! was sent away from the mill, because her master feared others might be tempted to follow her example. Are any words of condemnation needed for a system which makes the idea of suicide contagious?

